# Application of Plasma Sprayed Cu Intermediate Layers in the Soldering Process of Graphite Composite to 6060 Aluminum Alloy

**DOI:** 10.3390/ma13225114

**Published:** 2020-11-13

**Authors:** Tomasz Wojdat, Paweł Sokołowski, Leszek Łatka, Julia Chmielewska, Weronika Kurantowicz

**Affiliations:** Department of Metal Forming, Welding and Metrology, Faculty of Mechanical Engineering, Wroclaw University of Science and Technology, 50-370 Wroclaw, Poland; tomasz.wojdat@pwr.edu.pl (T.W.); leszek.latka@pwr.edu.pl (L.Ł.); 237392@student.pwr.edu.pl (J.C.); weronika.kurantowicz@gmail.com (W.K.)

**Keywords:** intermediate layer, graphite composite, soldering, 6060 aluminum alloy, plasma spraying, coatings

## Abstract

The work focuses on issues related to the soldering of graphite composite to 6060 aluminum alloy. The graphite composite is of great interest of the transportation industry as it is widely used in slides responsible for current collection from overhead catenary. The slides should meet various criteria resulting from relatively complex working conditions, e.g., in terms of electrical conductivity, self-lubricating, resistance to changing weather conditions, etc. Such an application has extensive requirements, mainly for a joint of graphite slide with aluminum body. The direct soldering of slide plates made of graphite composite to aluminum alloy collector head causes many technological problems and is not possible. In this study, the application of thin plasma sprayed (APS) copper intermediate layers is investigated for that purpose. After soldering process, the microstructural analysis confirmed the proper joint formulation, i.e., the soldering gap of 0.2 mm was well-filled with the solder over the entire width of joint. The soldered joints were then subjected for static shear testing. The obtained shear strength was in a relatively wide range of 13.04 to 20.50 MPa, which was influenced by various fracture mechanisms. Finally, the fact that reaction zones were not formed in investigated joints during soldering was confirmed by EDS analysis and micro hardness values, which were very similar to the ones of raw materials.

## 1. Introduction

An unceasing development in the area of machine building, oriented on improving quality and lower-priced operation, may be observed in some industries currently. One of these is the transportation, including public rail transport, where the requirements regarding the efficient operation, punctuality, and maintenance costs are of great importance. The rail vehicles are usually supplied with the electric power taken from the railway electrification system. Such a system includes very often overhead lines which make contact with an electric train equipped with a current collector, usually in form of a pantograph [1]. In order to achieve an efficient, constant, and high-speed current collection, the materials used for production of the overhead contact system (OCS) should meet various tribological, electrical, and mechanical criteria. One of the most critical element in a whole OCS is a slide that is directly pressed against the overhead lines. The application of graphite and graphite composite for slides is beneficial, as such materials are characterized by good electrical conductivity as well as appropriate mechanical and tribological performance, caused by low friction coefficient and self-lubricating properties [1,2,3].

However, the application of graphite and graphite composites is limited due to the difficulties with joining them to other materials, usually aluminum alloys. One of the possible ways is to use adhesives, but unfortunately such joints are not able to provide required electrical conductivity, even when using composite adhesives with different powder fillers [2]. In case of overhead contact systems this is not acceptable due to the large power losses generated and the resultant increased costs of rail operation. The sufficient electrical conductivity can be achieved by using methods that enable obtaining joints characterized by metallic continuity, like soldering or brazing [4].

The high temperature brazing processes, including vacuum ones, where diffusion phenomena have a main impact, may be also used for such joining. However, 6060 aluminum alloy is mainly used for the panhead, this is the body to which graphite-based slide plates are directly joined to. Such aluminum alloy is usually subjected to heat treatment (artificial aging), after which it should be not exposed to temperature higher than 200 °C in further processing [1]. Therefore, in order to meet the requirements set by pantograph manufacturers, it is necessary to develop a low temperature joining technology of graphite and its composites to 6060 aluminum alloy. This causes that the selection of materials which may be used for such joining process is also limited.

The trials with low temperature soldering processes showed that insufficient wettability by solders and lack of proper fluxes are among the main issues limiting the development of joining technology of mentioned materials [1,2]. Therefore, some of the solution to that problem may be the application of intermediate layers prior soldering. Such idea is known in joining technology and was applied already for example for brazing of copper to aluminum by using zinc filler metal or for brazing of cooper to 18/10 stainless steel by using copper phosphorus filler metal [5,6]. In both cases, the electrochemical routes were selected for the deposition of Zn-Ni and Ni-Cu interlayers [7,8]. Furthermore, Cu interlayer applied by electrochemical method was already considered for soldering of graphite composite to aluminum [2,9,10]. The coating was deposited on the surface of graphite composite, while joining process was carried out using the S-Sn60Pb40 filler metal and chemically active flux. This allowed to form a proper joint between the materials [10], having satisfactory quality and good mechanical properties [2,9]. The different thermal spray processes were also tested for that purpose. The basic ones, like flame and arc spraying enabled efficient deposition of metallic layer, e.g., Zn, Al, Al-Ni, or Ni-Al. Unfortunately, the relatively high in-flight oxidation of metal particles caused limited wettability of interlayer by most of fluxes [6,8]. Thus, a novel concept of low pressure cold spraying (LPCS) was investigated [8]. The limited in-flight temperature and much higher velocity of powder particles obtained during spraying allow obtaining interlayers characterized by negligible oxidation rate and low porosity [1,5,11,12,13,14,15,16]. LPCS coatings showed improved soldering properties and were successfully applied in soldering of dissimilar materials. However, as presented in [1], the Cu interlayer cannot be directly deposited on graphite or graphite composite by cold spray method. The copper particles are too hard and not enough plastic as for such substrate. When accelerated to supersonic speed they cause erosion of graphite rather than formulate a coating. In such a case it is necessary to first deposit a bond coat for Cu layer, made of softer and more plastic metal, aluminum for example. This makes the whole process longer and more complicated in terms of metallurgical reactions that take place later, during joining process itself. Furthermore, such a procedure results in higher porosity and roughness of Cu topcoat, which directly increases the number of pores and voids in a solder joint. As a result, the properties of graphite to 6060 aluminum alloy joint are of similar strength as the ones prepared with electrochemically deposited Cu interlayer [8].

In this work, an idea of using APS deposited Cu interlayers for the soldering of graphite composite to 6060 aluminum alloy is proposed. It is considered that plasma spraying may combine the advantages of thermal and kinetic spraying for that application, i.e., high temperature of jet that causes melting of particles and subsonic velocity of particles limiting the in-flight oxidation of copper powder particles [17,18,19].

## 2. Materials and Methods

### 2.1. Materials

**Figure 1:**
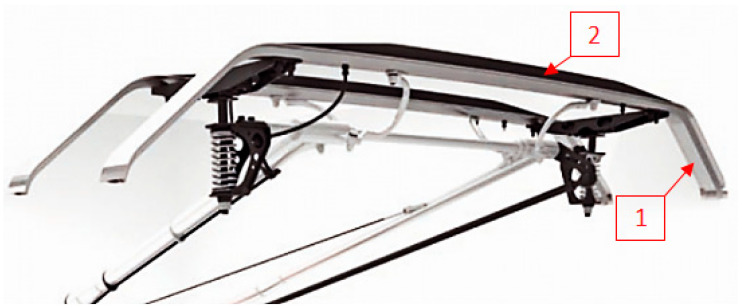
Conventional design of pantograph’s panhead: 1—panhead body (6060 aluminum alloy); 2—contact strips (graphite composite).

The elements to be welded are representative for pantograph construction (Figure 1). This means 6060 aluminum alloy, which is used for production of panhead and graphite composite as for slide plates [20].

Aluminum 6060 alloy is characterized by moderate ultimate tensile strength (Rm ≌ 190 MPa) and shows moderate fatigue properties. At the same time it has increased corrosion resistance and very good weldability. It may be easily cold worked as well. Alloy 6060 is a lightweight material, so it is usually used instead of steel in order to reduce the total weight of the structure, even up to 60%. It is also characterized by low melting point of 610–650 °C. Table 1 presents chemical composition of 6060 aluminum alloy. This was measured by X-ray fluorescence analyzer Fischerscope X-ray XDL-B (Fischer GmbH, Achern, Badenia-Wirtembergia, Germany) and then compared to EN 573-3:2014-02 standard [21].

**Figure 2:**
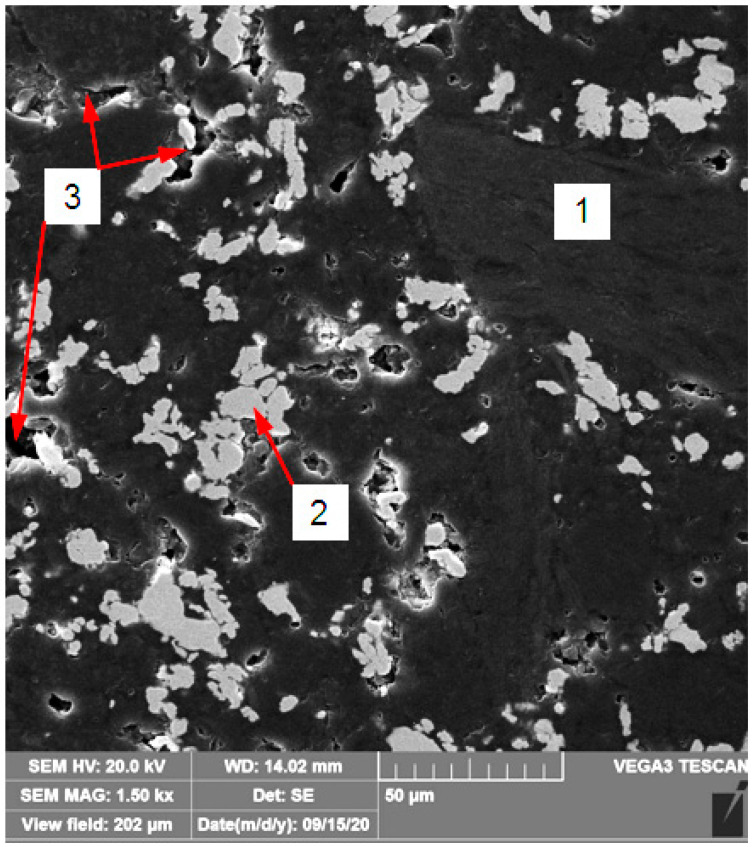
Graphite composite microstructure: 1—graphite matrix; 2—Cu powder (reinforcement); 3—pores.

On the other hand, the graphite composite used in the study is a mixture of pitch coke and graphite with addition of 40 wt % of copper powder (Figure 2). This was bonded with epoxy resin and then burned to a glassy carbon. The basic properties of such composite are as follows [1,10]:

1. shore hardness of about 90 oSh D (about 100 HV);
2. good tribological performance (wear rate about 2 × 10^−7^ mm^3^/Nm)-graphite acts as a lubricant;
3. high electrical conductivity;
4. total porosity up to 10%;
5. negligible liquid absorption.

The use of graphite composite for current collection in the overhead contact system is much more beneficial than previously used slide plates made of copper. The Cu contact strips had to be regularly lubricated, otherwise the catenary was damaged due to too high friction. A new concept of using graphite composites eliminates such risk mainly because of self-healing and self-lubricating properties. The lower susceptibility to damage caused by icing is another benefit of using such OSC elements [22].

**Figure 3:**
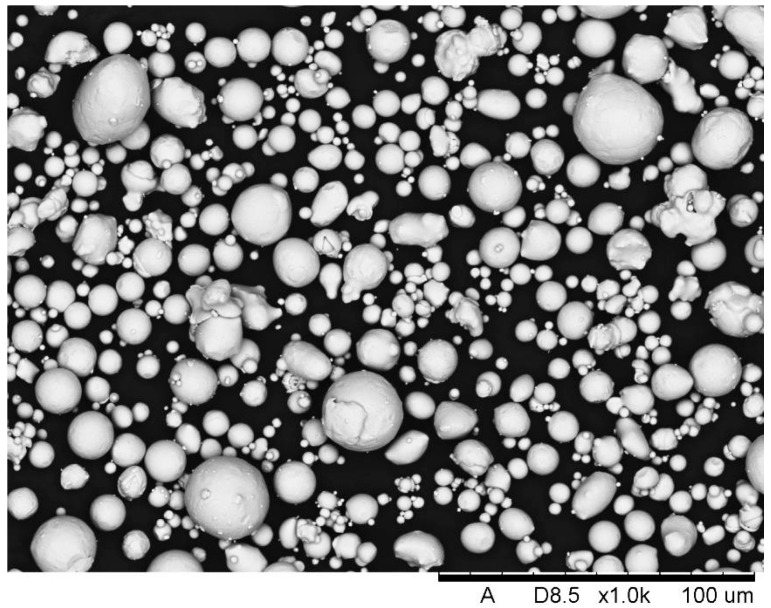
Spherical morphology of Cu powder that was used in APS process.

The copper interlayer was deposited by conventional atmospheric plasma spraying (APS) process. As a feedstock material a spherical Cu powder with particle size in a range −63 + 20 µm was used (Figure 3). The interlayer was sprayed onto graphite composite and 6060 aluminum alloy as well.

The S-Sn60Pb40 filler metal was used for soldering process with a melting temperature in range 183–190 °C [23] and diameter 2.5 mm. This filler metal was widely used in the electronics industry in the 90s, until its withdrawal under the RoHS Union Directive announced in July 2006. However, if the soldering temperature is maintained below 200 °C, as it was in this study, the use of tin–lead solders is still possible [16].

Furthermore, Stay Clean Aluminum flux was used during soldering process. This is a conventional flux applied when using tin or tin–lead solders. It was used to chemically clean the surfaces of materials and to ensure their good wettability with Sn-Pb filler metal.

The selection of filler metal, interlayer material and flux was caused by the maximum temperature limit for joining process, which was set as 200 °C. As mentioned above, such temperature is still acceptable in terms of not introducing significant microstructural changes and weakening the corrosion and mechanical properties to artificially aged 6060 aluminum alloy. The selected materials show relatively good weldability under low temperature soldering.

### 2.2. Joint Formulation

**Figure 4:**
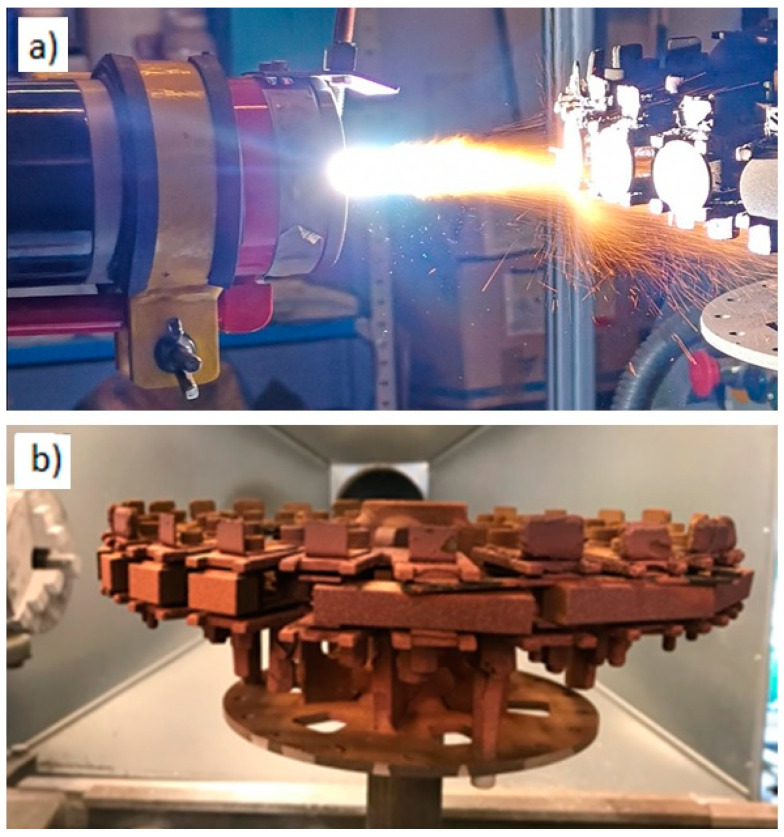
Robotized APS set-up: SG-100 plasma torch under operation (**a**), and sample holder mounted to a welding turntable (**b**).

Prior to the soldering of graphite composite to 6060 aluminum alloy it was necessary to deposit an intermediate layer of copper. This was done by well-established atmospheric plasma spraying (APS) process. The conventional one cathode and one anode SG-100 plasma torch (Praxair, Danbury, CT, USA) was used for Cu coating spraying. The Cu powder was injected radially to the plasma jet. The injector was mounted outside the plasma torch, to limit the thermal exposition of Cu particles to hot plasma gas (Figure 4a). In order to keep a high repeatability of deposition process the graphite composite and 6060 aluminum alloy substrates were fixed to a carousel. The sample holder was rotated by welding turntable with a constant speed, while the linear movement of the torch at a set stand-off distance of 90 mm was ensured by six-axis industrial robot FANUC R-2000iA (Fanuc EC S.A., Echternach, Luxenbourg). In such a way, 30 substrates were coated at once, so all samples underwent exactly the same spray conditions (Figure 4b).

The main parameters used for plasma spraying of Cu coatings are mentioned in Table 2. The pre-selection of spray variables was done first. This showed that the plasma electric power need to be relatively low in order to do not overheat Cu powders and decrease their oxidation in plasma jet. For that purpose, only Ar as a plasma forming gas and relatively short stand-off distance were used as well. All the substrates were sand-blasted (by 80 µm Al_2_O_3_) and cleaned in an ultrasonic bath in ethanol prior the intermediate layer deposition.

**Figure 5:**
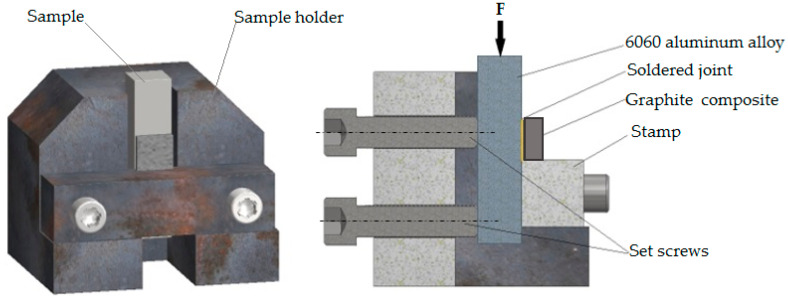
Sample holder used for static shear tests.

The geometry of graphite composite and 6060 aluminum alloy substrates were determined by sample holder used for static shear testing (Figure 5). Aluminum alloy samples were of 12 × 12 × 75 mm^3^ and graphite composite of 10 × 12 × 20 mm^3^.

After the Cu coating deposition the samples were joined together by using flame soldering. During the joining process, the soldering gap of 0.2 mm was used and was precisely set by using calibrated steel distance wires. Based on own experience, this size of soldering gap provides high capillary properties needed to efficiently spread the melt filler over the surfaces to be joined. Then, the samples were heated by propane–oxygen flame up to 200 °C, which is just above the melting temperature of flux and solder. In that soldering temperature the flux material (Stay Clean Aluminum Flux) was already activated, allowed reducing oxides formed on the both surfaces as well as improved filling of soldering gap by solder. The joining process was followed by the application of thin-lead solder (S-Sn60Pb40) to the soldering zone. The material was fed manually on one side of the soldering gap until it flew out on the opposite side. Then, the whole soldering gap was filled with the S-Sn60Pb40 solder and the joint was finally formed.

## 3. Results and Discussion

### 3.1. Metallography

**Figure 6:**
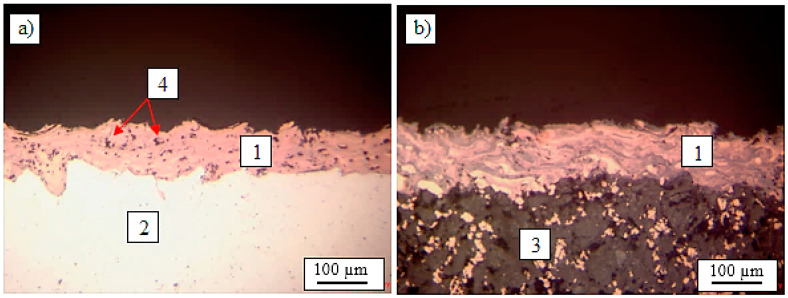
Plasma sprayed Cu coating on 6060 aluminum alloy (**a**) and on graphite composite substrate (**b**); 1—Cu coating; 2—6060 aluminum alloy; 3—graphite composite; 4—pores.

The samples were subjected to conventional metallographic preparation routine. After precise cutting, the samples were embedded in an epoxy resin. Afterwards, the samples were subjected to grinding and polishing procedure. The low magnification observations of samples was done by Nikon Eclipse MA200 (Nikon Imaging Japan Inc., Tokyo, Japan). The cross-section of Cu coating (1) deposited on aluminum alloy (2) and graphite composite substrates (3) are presented in Figure 6. It can be observed that the coating was well attached to the substrate surface in both cases and uniformly covered both substrates despite having a relatively low thickness as for the conventional plasma spraying. The substrate asperities created by sand-blasting are well coated with copper and the interface between the substrates and coating was free of any defects, like voids or cracks. Furthermore, the coating thickness seemed to be similar along the entire cross-sections. The measured thickness of coatings was in a range of 60 to 80 µm in case of 6060 aluminum alloy substrate and in range 70 to 90 µm in case of graphite composite substrate. The copper coatings contained some pores (4), which are mainly formed when gas is trapped between solidifying coating material. However, the distribution of pores at the coating cross-section is also quite uniform. It was observed that the porosity of Cu coatings was greater when using 6060 aluminum alloy substrates, which could be caused by its high affinity for oxygen, which in that way could influence the Cu deposition. Anyway, in both cases, the typical structure of plasma sprayed coatings was identified.

**Figure 7:**
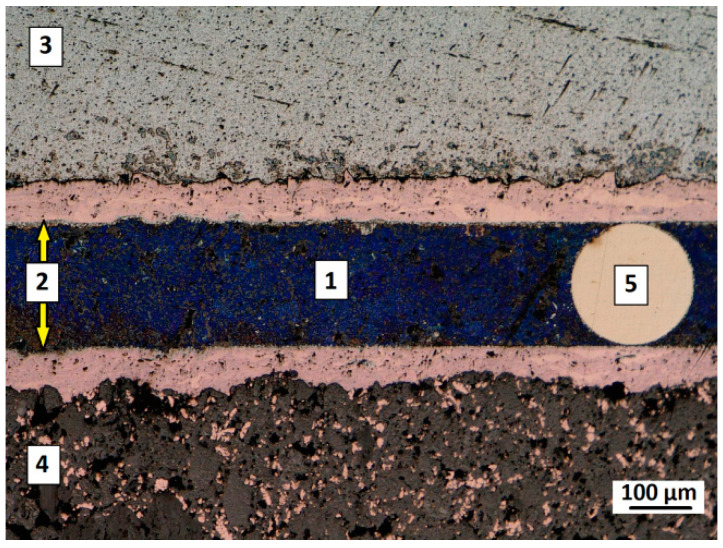
Cross-section presenting microstructure of soldered joints: 1—solder; 2—Cu coating; 3—6060 aluminum alloy; 4—graphite composite; 5—steel distance wire.

Afterwards, a similar microstructural investigations were carried out in case of soldered joint of 6060 aluminum alloy to graphite composite. As presented in Figure 7, the joint was formed properly and its quality is satisfactory. The soldering gap of 0.2 mm was well-filled with the solder over the entire width of joint (a steel distance wire is also visible on the joint cross-section). The solder incompatibilities were not observed along the cross-sections of soldered joints.

**Figure 8:**
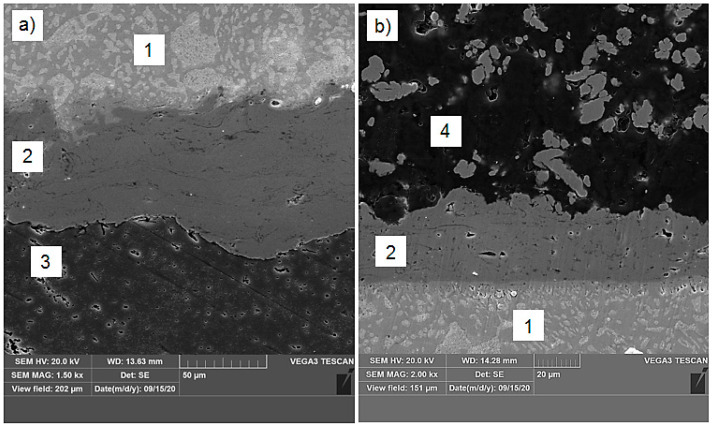
Interface between solder and Cu intermediate layer close to 6060 aluminum alloy (**a**) and close to graphite composite (**b**); 1—solder; 2—Cu coating; 3—6060 aluminum alloy; 4—graphite composite.

Then, the microstructural studies by scanning electron microscopy were carried out (Tescan Vega3, Tescan, Brno-Kohoutovice, Czech Republic). The observations were done under the secondary electrons. The cross-sectional SEM observations showed that Cu coatings were well built-up splat by splat, this means that the powder particles were well heated by plasma jet and then well flattened while hitting the surface. The non-melted initial Cu powder particles were not observed in the final coating structure. At higher magnification, the single and relatively fine pores inside Cu coatings were well visible, which is quite typical for APS deposits. Porosity was also observed in the structure of graphite composite. However, the pores were not formed in the solder layer. Therefore, the overall porosity in the soldering region may be assumed to be relatively low. The proper wetting of Cu coating by solder resulted in formulation of the adhesive-type of joint without visible reaction zones. The heating of substrate materials during soldering did not cause any delamination of intermediate layer on both, 6060 aluminum alloy and graphite composite, sides. The interface between Cu coating and solder was free of defects, as showed in Figure 8a (aluminum alloy side) and Figure 8b (graphite composite side).

**Figure 9:**
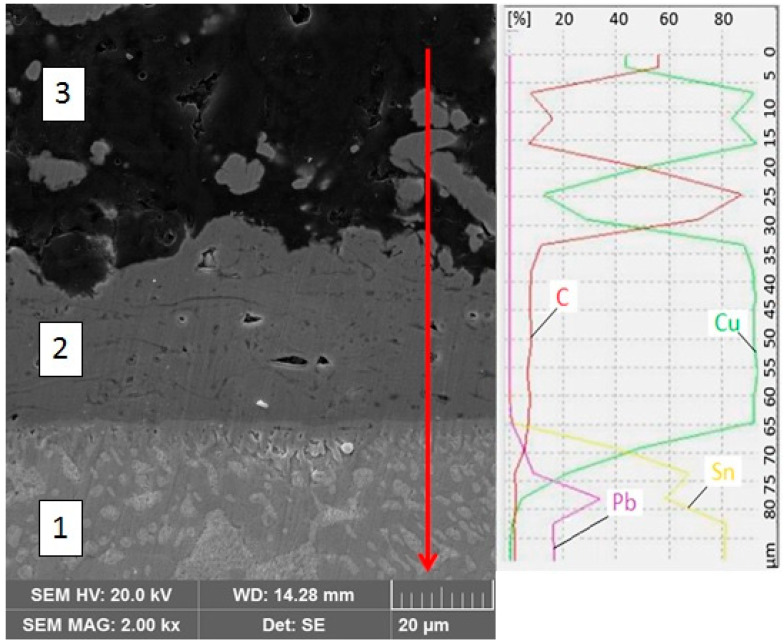
Micrograph of the interface between solder and Cu intermediate layer close to graphite composite together with linear EDS analysis: 1—solder; 2—Cu coating; 3—graphite composite.

As mentioned above, the reaction zones were not observed in the formulated joints. Linear EDS analysis (Figure 9, graphite composite side) showed sharp transitions of individual elements, characteristic for each zone of the joint. The base material, which is a graphite composite (3), contains carbon (C) and Cu reinforcement. Moving towards the joint, a 30 µm wide transition layer can be observed, which is a plasma sprayed Cu coating (2). Then, the solder layer (1) is made of dark α(Sn) + β(Pb) eutectic with bright β(Pb) precipitates.

**Figure 10:**
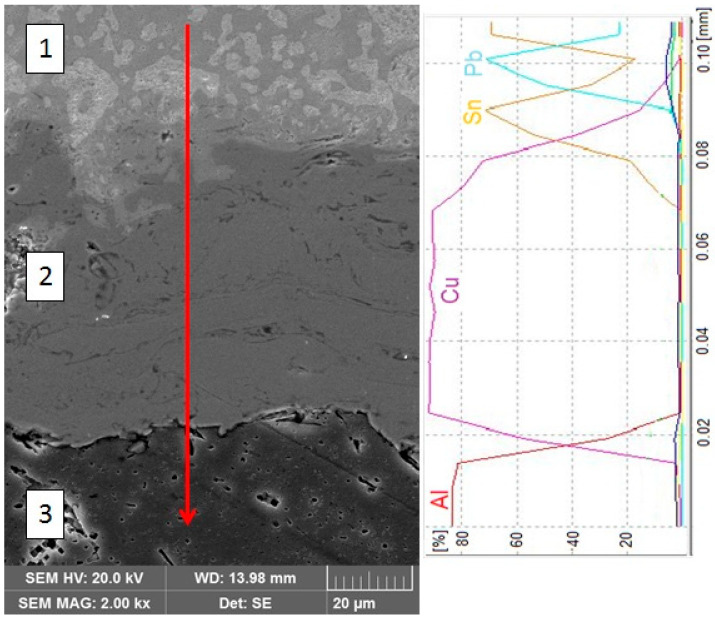
Micrograph of the interface between solder and Cu intermediate layer close to 6060 aluminum alloy together with linear EDS analysis: 1—solder; 2—Cu coating; 3—6060 aluminum alloy.

A similar findings were observed inside the joint on the 6060 aluminum alloy side (Figure 10). There is no visible diffusion zone in the solder layer (1) just above the Cu (2) coating. The solder is composed of dark α(Sn) + β(Pb) eutectic with bright β(Pb) precipitates.

### 3.2. Mechanical Properties of Soldered Joints

In order to determine the mechanical strength of graphite composite to 6060 aluminum alloy soldered joints the static shear test was performed. The studies were carried out by using universal testing machine Zwick/Roell Zmart-PRO (Zwick-Roell GmbH, Badenia-Wirtembergia, Ulm, Germany) with following testing parameters:

- test speed of 2 mm/min;
- load up to 10 kN.

As presented in Figure 5 above, the test sample was mounted in a dedicated holder that enabled to determine the shear strength under compression test. The geometry of samples was determined by a sample holder. After the mounting of the soldered parts, the side surface of graphite composite was supported by a stamp. During loading, the jaw clamps were pressing the aluminum alloy body, which resulted in shear stresses in the solder layer and caused the fracture of the soldered joint. Seven tests were conducted for the discussed samples and after fracture mode in each case was investigated.

In order to precisely calculate the shear strength, the exact joining area was measured under digital microscope Keyence VHX-6000 (Keyence Co., Osaka, Japan). This was influenced by the length of graphite composite substrate, as the 6060 aluminum alloy substrate was cut off from calibrated 12 mm rectangular bar. The results of static shear test are collected in Table 3. The calculated shear strength was in a relatively wide range of 13.04 to 20.50 MPa, which was caused by the different fracture mechanisms observed after testing.

**Figure 11:**
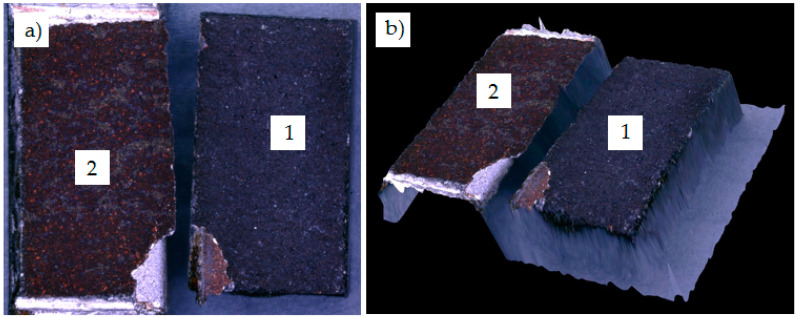
Adhesive fracture in solder joint under shear test (sample no. 2) (**a**) and 3D view of fracture (**b**); 1—graphite composite; 2—Cu coating on 6060 aluminum substrate.

In the case of sample no. 2, with the lowest shear strength (13.04 MPa), the adhesive fracture occurred. The Cu coating was fully pulled off of the graphite composite substrate (Figure 11). Moreover, the intermediate layer did not undergo decohesion or fragmentation during testing and together with solder was still bonded to 6060 aluminum alloy substrate.

**Figure 12:**
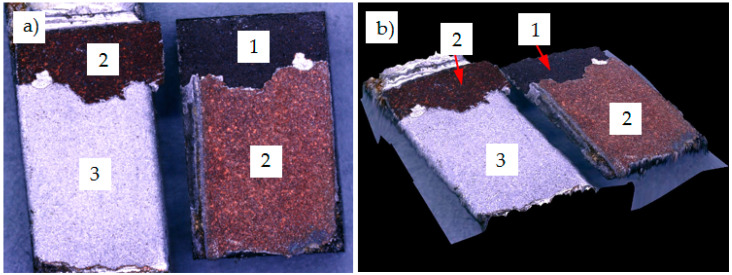
Adhesive fracture in solder joint under shear test (sample no. 4) (**a**) and 3D view of fracture (**b**); 1—graphite composite; 2—Cu coating; 3—6060 aluminum alloy.

The opposite effect was observed in sample no. 4, which has damaged at highest shear force (resulting in shear strength of 20.50 MPa). The fracture mechanism was also clearly adhesive but in this case the Cu coating was debonded from the aluminum alloy substrate (Figure 12). A similar mechanism was observed in sample no. 1, so the one showing the second highest shear strength (19.2 MPa) among all tested soldered joints.

**Figure 13:**
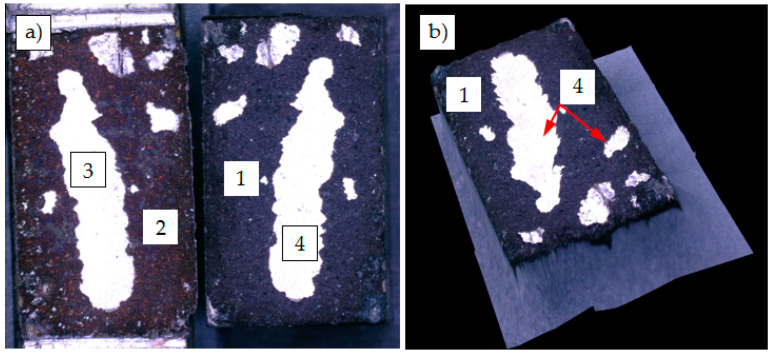
Mixed solder/intermediate layer fracture under shear test (sample no. 5) (**a**) and 3D view of graphite composite fracture (**b**); 1—graphite composite; 2—Cu coating detached from the graphite composite; 3—solder; 4—Cu coating detached from the solder.

In other cases, this means samples no. 3, 5, and 7 the shear strength was in a medium range of 14.28 to 16.88 MPa. The fracture mechanism was in these cases the mixed (see Figure 13), adhesive-cohesive, one. The main part of Cu coatings was pulled off of the graphite composite substrate whereas some smaller area was debonded from the solder layer. Therefore, the adhesive fracture prevailed and then determined the overall shear strength of the soldered joint.

**Figure 14:**
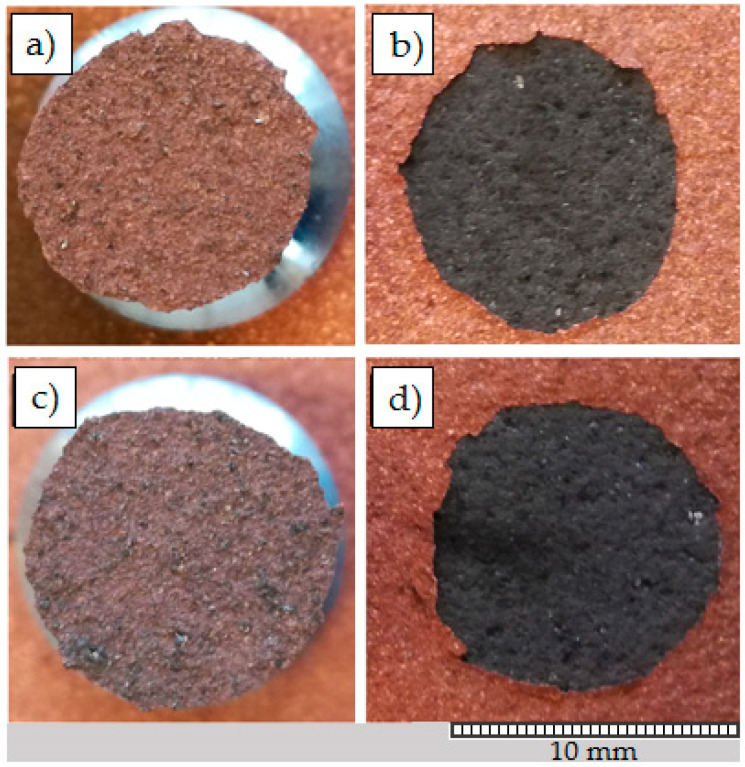
Surfaces of corresponding test dollies (**a**,**c**) and graphite composite substrates (**b**,**d**) after pull-off test.

The presented results showed that the adhesion of Cu to graphite composite is one of the key factor influencing the final strength of soldered joint. For that reason, the bond strength of Cu coating to graphite composite substrate was tested according to the ASTM C633-13 standard [24]. The test dolly was glued to Cu coating by using epoxy adhesive distal classic having the average strength of 50 MPa itself. The test dolly was of 10 mm in diameter. The test dolly was then pulled off of the substrate by automatic adhesion gauge Electrometer 510 (Elcometer, Manchester, UK) with the load rate of 0.4 MPa/s. Five samples were tested in total and the average adhesion of coatings was equal to 7.5 MPa. The surfaces of the test dolly and substrate were observed to determine the fracture type, the examples are presented in Figure 14. The adhesive type of fracture was observed clearly after each test. In some cases, the single graphite composite particles were ripped out from the substrate and are visible on the test dolly surface in form of dark spots (Figure 14c).

It should be noticed that the results of shear strength of investigated soldered lap joints with plasma sprayed Cu intermediate layers were already much higher than in previous studies [2,15,16]. The application of galvanic Cu coating in similar lap joints resulted in shear strength in range of 6.3–17.6 MPa, depending on the substrate surface pretreatment method. When using novel low pressure cold spray (LPCS) process for a deposition of Cu intermediate layer the maximum shear strength did not exceed 10 MPa [1]. However, as in plasma spraying, there are several parameters to be optimized, there is still a possibility to increase mechanical properties of presented joints. The one to be investigated first in further studies is the graphite composite treatment prior plasma spraying.

Finally, the measurements of hardness were performed, both in raw materials as well as on the cross-section of the entire joint. For that purpose the precise instrumental indentation tests (IIT) have been performed with the use of NHT3 nanoindenter (Anton Paar, Graz, Austria). The procedure was selected based on ATSM E2546 standard [25] and the Berkovich indenter was used here. In order to have some comparison with classical microhardness measurements, the maximum load (P_max_) value used in IIT was equal to 250 mN. The loading and unloading force was two times greater than the value of Pmax and the dwell time was equal to 15 s. The 10 imprints were made each time, which were used to calculate the average hardness and the standard deviation.

The hardness of as-sprayed Cu coating slightly differed, depending on substrate material. It was equal to 125 ± 6 HV 0.025 for coatings sprayed onto 6060 aluminum alloy and 106 ± 15 for coatings deposited onto graphite composite. In the literature, mostly multi-material plasma sprayed coatings containing copper—such as Cu-Ni, W-Cu, etc.—were analyzed [26,27]. Even if some works focuses on pure Cu thermally sprayed coatings, a different substrate materials were selected. For example, as showed in [28], the hardness of Cu coatings sprayed onto 316L stainless steel substrate was in the range of 144–165 HV 0.1, depending, e.g., on the spray distance. Ranjan et al. [29] studied pure plasma sprayed coatings deposited onto Cu substrates. It their work, the microhardness was between 97–120 HV and was dependent on plasma power. However, regardless the spray set-up arrangements and deposition parameters, the substrate properties may significantly influence the growth of coating and then, its hardness. Trompetter et al. [30] showed that substrate hardness is one of the important factor in thermal spraying that should be considered for controlling proper deformation of powder particles upon hitting the substrate material. The greater heat energy being generated by plastic deformation on harder substrates enables formation of splats, higher packing of coating and then the increase in hardness of coating material is observed. After soldering process, some differences were observed in the hardness of Cu coatings. On the side of aluminum substrate, the hardness of Cu coating slightly increased to 136 ± 6 HV 0.025, while on the graphite composite side decreased to 86 ± 12 HV 0.025. Although the mechanical properties of the coatings slightly varied after the soldering process, this was rather not influenced by microstructural effects, as described above. However, this should be analyzed in more detail in further works, in order to determine how the soldering conditions influence the local hardness in each material. The hardness of substrate materials was the same, as measured before and after soldering process. This was equal to 113 ± 7 HV 0.025 in case of aluminum alloy, 213 ± 6 HV 0.025 in case of copper reinforcement and 26 ± 2 HV 0.025 in case of graphite matrix. In case of solder layer, the average hardness was equal to 14 ± 2 HV 0.025, which is characteristic for Sn-based solder.

## 4. Conclusions

The presented study shows that there is a possibility to join 6060 aluminum alloy to graphite composite by means of low temperature soldering. This kind of material combination is very popular in pantograph construction (joining of slide plates to the panhead).

For that purpose, the Cu plasma sprayed intermediate layer was tested in this work. In order to avoid difficulties with solderability of the two mentioned materials, both of them were coated first with 60–90 µm thick copper coating. Then, the materials were joined together by using flame soldering. Stay Clean Aluminum flux and S-Sn60Pb40 solder were used for that purpose.

The quality of joints was very high and the microstructural investigations showed that the joints are free of solder incompatibilities, like porosity or gas pores. At the interface between solder and Cu intermediate layer, close to 6060 aluminum alloy and close to graphite composite, the reaction zones were not observed.

The shear strength of soldered lap joints was up to 20 MPa and was determined mostly by the adhesion of Cu coating to graphite composite substrate. This makes it possible to still optimize the plasma spray parameters. However, the results are already satisfactory, as the shear strength values are even 100% higher than in similar joints, where Cu intermediate layer was applied by low pressure cold spray and at least 15% higher than in case of galvanic Cu intermediate layer.

The hardness of the as-sprayed Cu coatings was dependent on substrate material. The slightly higher hardness was obtained for harder 6060 aluminum alloy substrate material than in case of graphite composite. As a result of the soldering process, the hardness of the Cu coatings was slightly changed. From the side of the aluminum substrate, the hardness increased; while from the side of the graphite composite, the hardness decreased. This seems that soldering process for Cu coatings is a kind of heat treatment that may cause slight changes in the mechanical properties of the coatings. However, the microstructural effects were not observed by SEM and EDS analysis. This will be studied in more detail in future works.

## Figures and Tables

**Table 1:** Chemical composition of 6060 aluminum alloy.

Range or Maximum Content of Elements, wt % [21]								
Mg	Si	Cu	Mn	Fe	Cr	Zn	Ti	Al
0.35–0.60	0.3–0.6	max 0.1	max 0.1	0.1–0.3	max 0.05	max 0.15	max 0.1	balance
**Spectral Analysis of Elements, wt %**								
0.53	0.39	0.06	0.09	0.14	0.04	0.11	0.05	balance

**Table 2:** Plasma spray parameters of Cu interlayer.

Spray Parameter	Value
Plasma gas flow rate (Ar)	45 L/min
Carrier gas flow rate (Ar)	3 L/min
Cu powder flow rate	20 g/min
Electric power	20 kW
Spray stand-off distance	90 mm
Torch scanning velocity	400 mm/min

**Table 3:** Results of static shear test of soldered joints.

Sample No.	Area (mm^2^)	Shear Force (N)	Shear Strength (MPa)
1	152.65	2950	19.32
2	153.65	2000	13.04
3	154.07	2600	16.88
4	151.23	3100	20.50
5	154.07	2200	14.28
6	151.94	2300	15.14
7	155.49	2500	16.08
